# Ubiquitous mitochondrial creatine kinase promotes the progression of gastric cancer through a JNK-MAPK/JUN/HK2 axis regulated glycolysis

**DOI:** 10.1007/s10120-022-01340-7

**Published:** 2022-09-16

**Authors:** Yushuai Mi, Quanhui Li, Bingtian Liu, Dehai Wang, Ziping Liu, Tianshi Wang, Yuan Wang, Yifeng Zang, Yan Zhou, Yugang Wen, Yinlu Ding

**Affiliations:** 1 Department of Gastrointestinal Surgery, The Second Hospital, Cheeloo College of Medicine, Shandong University, No. 247 Beiyuan Street, Jinan, 250033 China; 2 Department of General Surgery, Shanghai General Hospital, School of Medicine, Shanghai Jiaotong University, 85 Wujin Road, Shanghai, 200080 China

**Keywords:** uMtCK, Gastric cancer, Prognosis, Glycolysis

## Abstract

**Background:**

Ubiquitous mitochondrial creatine kinase (uMtCK) transfers high-energy phosphates from mitochondrially generated ATP to creatine to generate phosphocreatine. uMtCK overexpression has been reported in several malignant tumors, however, the clinical significance and impact of uMtCK in gastric cancer (GC) has not been comprehensively studied.

**Methods:**

We first examined uMtCK expression in GC by quantitative real-time PCR and western blot assays. Then the clinicopathological significance of aberrant uMtCK expression was determined by immunohistochemical staining in a GC tissue microarray. Kaplan–Meier analysis was used for survival analysis. The biological functions of uMtCK in GC cells were explored by wound-healing, transwell assays and glucose metabolism assays in vitro as well as a liver metastasis model by spleen injection in nude mice in vivo.

**Results:**

We verified that the expression of uMtCK was substantially elevated in GC tissues, significantly associating with a poorer prognosis in GC patients, especially for those with advanced stage. In univariate and multivariate analyses, uMtCK expression emerged as an independent prognostic factor for both disease-free survival and overall survival. Functionally, we demonstrated that uMtCK promoted glycolysis in GC cells and facilitated their migration, invasion and liver metastasis in vitro and in vivo. Mechanistically, uMtCK enhanced GC progression in a HK2-dependent glycolysis via acting the JNK-MAPK/JUN signaling pathway.

**Conclusions:**

uMtCK could serve as a novel independent prognostic biomarker as well as potential therapeutic target for GC patients, particularly for GC patients with an advanced UICC stage and tumor recurrence.

**Supplementary Information:**

The online version contains supplementary material available at 10.1007/s10120-022-01340-7.

## Introduction

A steady decline in gastric cancer (GC) incidence and mortality rates has been observed recently. However, GC remains the third leading cause of cancer-related deaths worldwide [1, 2]. Despite advances in treatment, the overall prognosis remains poor due to tumor relapse and metastasis as well as a lack of effective molecular biomarkers to better identify prognostic factors and assist in the use of individualized therapeutic regimens [3, 4]. Therefore, there is an urgent need for the identification of novel prognostic and therapeutic markers to improve clinical outcomes in GC at advanced stage [3].

Previously, we demonstrated that Ras association domain family member 6 (RASSF6) acted as a tumor suppressor in GC and after up-regulated the expression of RASSF6 in GC cells, we identified that ubiquitous mitochondrial creatine kinase (uMtCK) was significantly decreased in GC cells compared with control groups using a loss of heterozygosity (LOH) analysis and cDNA microarrays [5, 6]. uMtCK is a member of the creatine kinase (CK) family, which is responsible for the transfer of high-energy phosphate from mitochondria to the cytosolic carrier, creatine [7]. Encoded by genes on human chromosome 15, uMtCK is synthesized by ribosomes in the cytosol and then translocated to the mitochondria [7, 8], localizing at the outer surface of the inner mitochondrial membrane [9]. Recently, some researches have demonstrated the overexpression of uMtCK in several malignant tumors, denoting a poor prognosis [10–12]. In breast cancer, the elevated expression of uMtCK showed a significant association with both a short disease-free survival (DFS) and an increased risk of relapse, serving as an independent prognostic marker for breast cancer [10]. Moreover, its overexpression promotes tumor growth by inhibiting apoptosis in tumor cells by down-regulating mitochondrial apoptotic pathway proteins [11]. Reduced uMtCK expression in HCC cells leads to inhibition in cell proliferation, migration and invasion [12]. However, to date, there are no reports regarding the clinic-pathological significance of uMtCK and its prognostic value in GC.

Deregulated or altered energy metabolism has been recognized as one of the “hallmarks of cancer” [13]. The existence of a link between aerobic glycolysis and tumorigenesis has been known for several decades ever since the “Warburg effect” [14]. Aerobic glycolysis is universally involved in rapid ATP synthesis [14], meanwhile numerous previous studies have reported that uMtCK together with cytosolic CK is essential for maintaining the normal function of cells, possibly through a continuous delivery of high-energy phosphates to the site of ATP utilization [15, 16]. Although the essential role of aerobic glycolysis in cancers has been widely investigated, the involvement of uMtCK in glycolysis in GC remains to be elucidated.

In the present study, we verified that uMtCK expression is up-regulated in GC, and aberrant uMtCK overexpression was associated with GC progression using a tissue microarray (TMA) with the data from 264 patients. In vitro and in vivo functional assays demonstrated that uMtCK promoted glycolysis in GC cells and further promoted their migration, invasion and liver metastasis. Moreover, our study on the mechanism confirmed that the JNK-MAPK/JUN/HK2 signaling pathway was involved in uMtCK-induced GC progression, offering a novel prognostic biomarker in GC patients after radical surgery.

## Materials and methods

Details are described in the Supplementary materials and methods.

## Results

### High uMtCK expression in GC tissues predicted a poor prognosis in GC patients

**Fig. 1:**
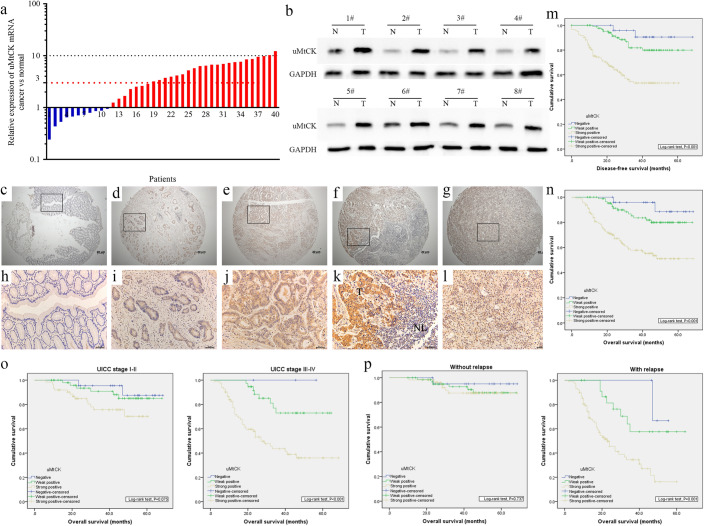
uMtCK is overexpressed in GC patients, predicting poorer prognosis, particularly for GC patients with advanced UICC stage and relapse. **a** Relative uMtCK gene expression in a series of 40 matched GC tissue specimens (*T*) and paired non-neoplastic gastric mucosal tissues (*N*). A logarithmic scale of the 2^–ΔΔCT^ was used to represent the fold change in the qPCR detection. **b** uMtCK protein expression was evaluated in 8 representative paired GC tissues by WB analysis, and GAPDH was the loading control. **c**, **h** Normal gastric epithelium (Negative staining); **d**, **i** well-differentiated GC tissues (Weak staining); **e**, **j** moderately differentiated GC tissues (Moderate staining); **f**, **k** *T*: poorly differentiated GC tissues (Strong staining); *NL*: normal lymph node tissues (Negative staining); **g**, **l** metastatic lymph node tissues (Strong staining). uMtCK protein was significantly higher in the GC tissues compared with the adjacent non-neoplastic gastric mucosal tissues, and uMtCK staining was mainly observed in the cytoplasm of the GC cells. Kaplan–Meier survival curves of patients with GC according to uMtCK expression. Patients with uMtCK-positive expression showed an obvious poorer disease-free survival (**m**) and overall survival (**n**) rate than patients with negative expression (*P* < 0.001 for both, log-rank test). Comparisons of the overall survival between the uMtCK-negative and uMtCK-positive expressing samples in the early UICC stage (I–II) cohort, in the advanced UICC stage (III–IV) cohort (**o**) and in patients with or without relapse (**p**). The *P* values were calculated by a log-rank test, and *P* < 0.05 indicates significance

To explore uMtCK expression levels in GC, we first analyzed uMtCK expression in two independent public datasets from Oncomine [17, 18], and found that the mRNA expression level of uMtCK was elevated in a majority of the GC tissues compared with that in adjacent non-neoplastic gastric mucosal tissues (Supplementary Fig. S1a, b). Then, we further evaluated the expression levels of uMtCK in 40 paired fresh specimens, finding that 29 (72.5%) of the GC tissues showed elevated uMtCK expression at the mRNA level compared with the matched adjacent non-neoplastic gastric mucosal tissues (Supplementary Table S4, Fig. 1a), which was consistent with the data from the public datasets. WB analysis further confirmed that uMtCK was overexpressed in the GC tissues, which were randomly selected from the upper 40 paired specimens (Fig. 1b). These results suggest that both at the mRNA and protein levels, the expression of uMtCK is higher in GC tissues than in their adjacent non-neoplastic gastric mucosal tissues.

To further investigate the relationship between uMtCK expression and GC clinical factors, IHC staining was used to detect uMtCK protein expression in a TMA, which contained 264 cases of primary GC paired with adjacent non-neoplastic gastric mucosal tissues and 104 lymph node metastasis (LNM) samples (Supplementary Table S5). uMtCK was distributed all across the cell body, except the nucleus, and representative IHC staining of uMtCK in the GC tissue and the normal mucosa is summarized in Fig. 1c-–l. Among the 264 non-neoplastic gastric mucosal tissues, 78.4% (207/264) showed negative uMtCK expression. However, uMtCK was prominently expressed in a majority of the GC specimens, showing a strong staining in 55.3% (146/264) of the specimens, weak staining in 35.2% (93/264) of the specimens and negative staining in only 9.5% (25/264) of the specimens. These results further confirmed that uMtCK expression was apparently up-regulated in GC tissues compared with that in adjacent non-neoplastic gastric mucosal tissues. More importantly, in the 104 available LNM tissues, 83.7% (87) of the specimens showed strong positive staining (Fig. 1g, l), which indicates that uMtCK overexpression may play a significant role in GC metastasis.

The relationship between uMtCK expression and clinicopathological characteristics are presented in Supplementary Table S6. The overexpression of uMtCK is highly correlated with tumor invasion (pT stage, *P* = 0.03), LNM (pN stage, *P* < 0.001), distant metastasis (M stage, *P* = 0.015), advanced UICC (Union for International Cancer Control) stage (*P* < 0.001), histological differentiation (*P* < 0.001) and tumor relapse (*P* < 0.001). The comparison of uMtCK expression in the primary GC tissues and the paired LNM samples indicated that uMtCK expression was strikingly elevated in the LNM samples (83.7%, 87/104 *vs.* 46.2%, 48/104; *P* < 0.001). However, no significant associations between uMtCK expression and age, gender, tumor location, tumor size, vessel invasion or nerve invasion were found in the present study (*P* > 0.05 for all, Supplementary Table S5).

To explore the role of uMtCK in GC patient survival, a Kaplan–Meier analysis with a log-rank test for DFS and OS was first conducted to evaluate the predictive role of uMtCK in GC patients using data from The Cancer Genome Atlas (TCGA). We found that uMtCK expression at the RNA level was only significantly associated with OS at the upper-tertile classification (Supplementary Fig. S1h). Subsequently, a Kaplan–Meier analysis with a log-rank test for DFS and OS was further used to evaluate the predictive role of uMtCK in GC patients based on our IHC staining data. Patients with tumors showing a higher uMtCK expression had a poorer prognosis than those with a lower uMtCK expression, which was demonstrated by the DFS rate and the OS rate (*P* < 0.001 for both, Fig. 1m, n). Then, in the univariate Cox regression analyses of the DFS and OS, T stage, N stage, M stage, UICC stage, differentiation, tumor relapse and uMtCK expression were significantly associated with both DFS and OS (Supplementary Table S7, S8). Furthermore, a multivariate analysis, which was used to analyze the parameters possessing significance in the univariate analyses, demonstrated that uMtCK expression remained an independent prognostic factor for both DFS and OS (Supplementary Table S7, S8), indicating that high uMtCK expression was an independent prognostic biomarker for GC.

To confirm the association between elevated uMtCK expression and tumor metastases or local relapse, regardless of the clinical stage, we further performed an OS analysis according to the UICC stages and tumor relapse. Notably, in patients at III–IV stage, uMtCK expression exhibited a significant association with a poorer OS, while in patients at I-II stage, uMtCK expression did not significantly affect OS (*P* < 0001 vs.* P* = 0.075, Fig. 1o). Furthermore, there was a trend towards a shorter OS in the patient group with uMtCK-positive tumors compared with the patient group with uMtCK-negative tumors with or without relapse (*P* = 0.001 *vs. P* = 0.737, Fig. 1p). Collectively, these data demonstrate that uMtCK overexpression could serve as a novel independent prognostic biomarker for a shorter OS independent of advanced clinical stage and tumor relapse.

### uMtCK overexpression facilitates GC cell migration, invasion and liver metastasis in vitro and in vivo

As we have demonstrated that high uMtCK expression positively correlated with GC recurrence and metastasis, therefore, we explored whether uMtCK accelerated GC cell metastasis functionally. We first evaluated uMtCK expression in seven GC cell lines and one normal gastric mucosa cell line, finding that the expression level of uMtCK was higher in all GC cell lines than in normal gastric cell line (Supplementary Fig. S2a). Then, small interfering RNA (siRNA 1#, 2#, 3#) specially targeting uMtCK and pcDNA3.1-uMtCK plasmid for gene overexpression were transfected in AGS/MKN28 cells, which showed the lower uMtCK expression and SGC-7901/MGC-803 cells, which had the higher uMtCK expression, with their efficiencies were confirmed by qPCR and WB, respectively (Supplementary Fig. S2b, c). Finally, we generated stable uMtCK overexpressing cell line (AGS/Lv-uMtCK) and knockdown cell line (SGC-7901/si-uMtCK-3#) for further study.

**Fig. 2:**
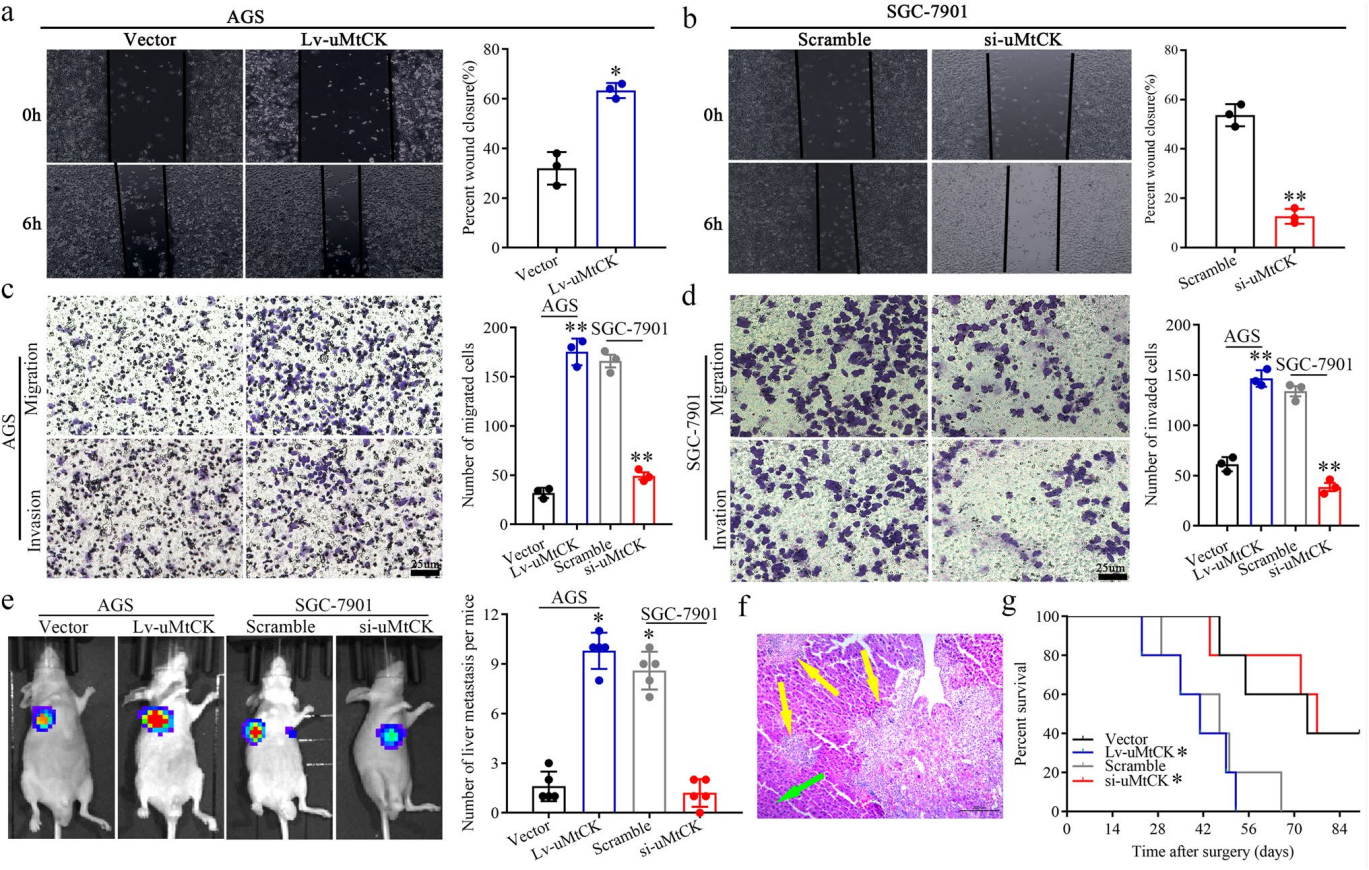
uMtCK facilitates GC cell migration, invasion in vitro and liver metastasis in vivo. uMtCK overexpression or knockdown increased or decreased GC wound-healing (**a**, **b**), migration and invasion (**c**, **d**) compared with their control group, respectively. **e–g** The impact of uMtCK on GC cells liver metastasis in vivo. Representative formation of liver metastases by a spleen injection of AGS/Lv-uMtCK-luc and SGC-7901/si-uMtCK–Luc as well as their control group cells into nude mice, respectively. **e** Representative images of the luciferase signals (*n* = 5). The number of liver metastatic lesions was counted (*P < 0.05). **f** The images of the liver metastatic lesions by HE (green arrows, normal tissues; yellow arrows, liver metastatic lesions). **g** OS of each group of mice injected with engineered cells. (*P < 0.05, *P < 0.01)

Wound-healing assays showed that uMtCK overexpression in AGS cells resulted in a rapid wound closure (Fig. 2a), whereas knocking down uMtCK expression in the SGC-7901 cells resulted in delayed wound closure, respectively (Fig. 2b). In addition, transwell assays demonstrated that ectopic overexpression or knockdown of uMtCK dramatically enhanced or impeded the number of GC cells migration and invasion, respectively (Fig. 2c, d). Furthermore, we investigated the functional impact of uMtCK on liver metastasis of GC cells in vivo. Stable AGS/SGC-7901 cells or control groups transfected with lenti-LUC virus were transplanted into nude mice via spleen injection and HE staining was used to count metastatic foci in mouse livers, respectively. We found that overexpression or knockdown of uMtCK increase or decrease the number of liver metastatic lesions in nude mice, respectively (Fig. 2e, f). uMtCK overexpression group or knockdown group compared with control groups, respectively (Fig. 2g). Accordingly, the above data indicated that high uMtCK expression could promote GC cells migration and invasion in vitro as well as liver metastasis in vivo.

### uMtCK enhances GC cell glycolysis in an HK2-dependent manner

**Fig. 3:**
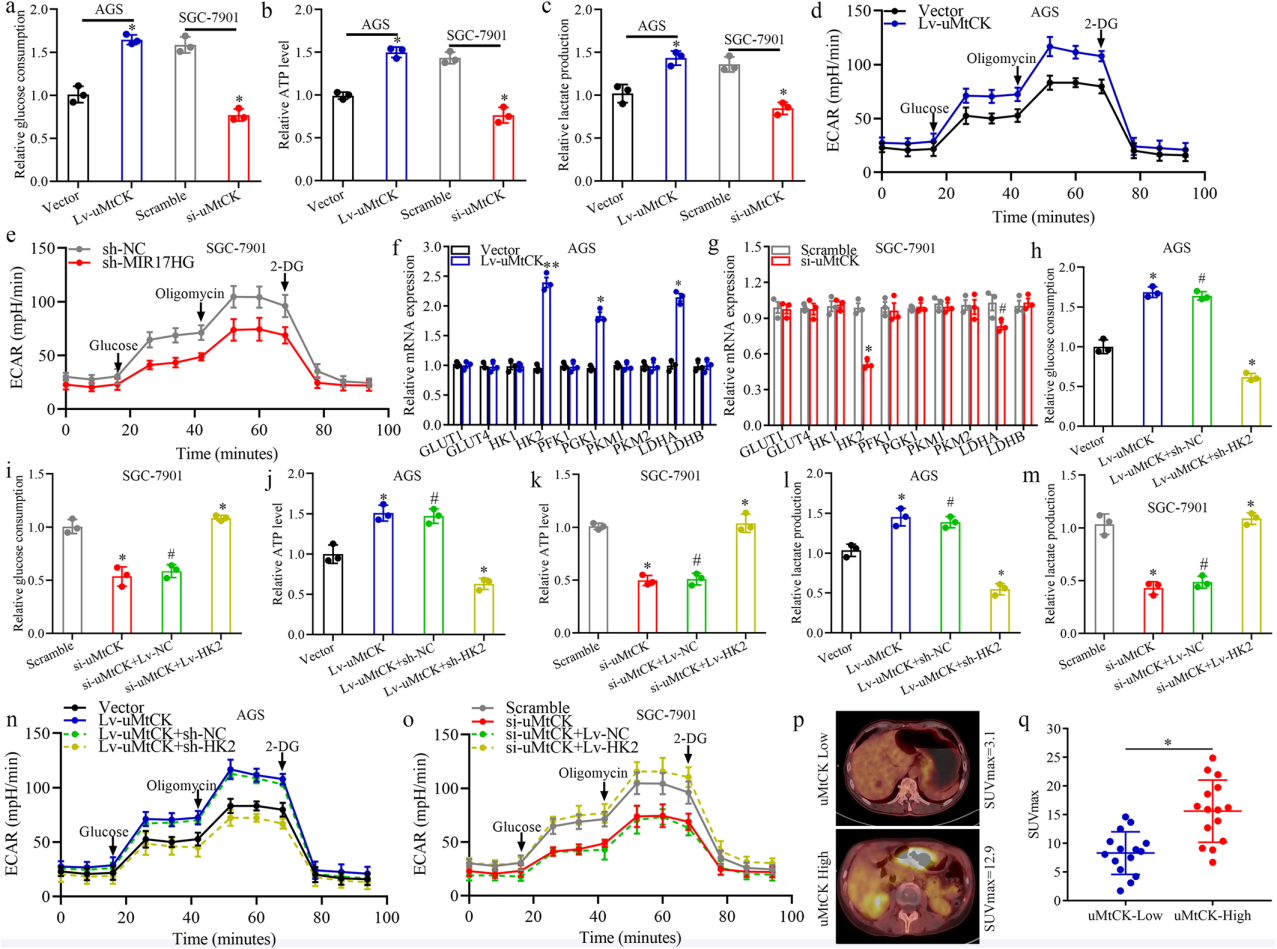
uMtCK promotes GC cell glycolysis in an HK2-dependent manner. **a** The effect of uMtCK overexpression or knockdown on changes in relative glucose consumption (**a**), ATP levels (**b**), lactate production (**c**) and ECAR levels (**d**, **e**) in GC cells AGS/Lv-uMtCK and SGC-7901/si-uMtCK as well as their control groups, respectively. The mRNA levels of a series of glycolysis-associated genes in uMtCK-overexpressing AGS (**f**) and in uMtCK-knockdown SGC-7901 cells (**g**), respectively. The effect of uMtCK overexpression or knockdown combined with HK2 knockdown or overexpression on the changes in relative glucose consumption (**h**, **i**), ATP levels (**j**, **k**), lactate production (**l**, **m**) and ECAR levels (**n**, **o**) in GC cells AGS/Lv-uMtCK and SGC-7901/si-uMtCK as well as their control groups, respectively. **p** Representative 18F-FDG PET/CT images of GC patients with low or high uMtCK expression. **q** Difference analysis of SUVmax in the uMtCK-low and uMtCK-high groups. (*P < 0.05, ^#^*P* > 0.05)

The “Warburg effect” in tumor cells promotes the glucose uptake, glycolysis, and pyruvate metabolism into lactic acid to produce energy under aerobic conditions and enhanced aerobic glycolysis is the primary feature of metabolic reprogramming in cancers that has been generally reported in GC [16, 19]. Interestingly, a gene set enrichment analysis (GSEA) based on TCGA database showed that elevated expression of uMtCK was positively with glycolysis/gluconeogenesis (Supplementary Fig. S3a) and we observed that the culture medium turned yellow faster in AGS/Lv-uMtCK cells than in SGC-7901/si-uMtCK-3# cells, indicating that uMtCK may promote acidification by increasing glycolysis in GC. To address this question, we further investigated the role of uMtCK on aerobic glycolysis of GC cells. We found that overexpression or knockdown of uMtCK significantly increased or inhibited glucose consumption, ATP and lactate production in GC cells, respectively (Fig. 3a–c). Furthermore, the measurements of extracellular acidification rate (ECAR) were executed to explicit the effect of uMtCK on aerobic glycolysis in GC cells. Consistent with the above results, GC cells with uMtCK overexpressing or knockdown displayed an enhanced or weakened glycolysis phenotype, respectively (Fig. 2d, e). Taken together, these results indicated that uMtCK strengthened aerobic glycolysis in GC cells.

As glucose transporters (GLUT1/4) as well as glycolytic enzymes, including hexokinase (HK1/2), phosphofructokinase 1 (PFK1), phosphoglycerate kinase 1 (PGK1), pyruvate kinase (PKM1/2) and lactate dehydrogenase (LDHA/LDHB), are critical regulators of the glycolytic pathway [20], we then investigated the expression profiles of these genes in GC cells with uMtCK overexpressing or knockdown, respectively. As shown in Fig. 3f, g, overexpression or knockdown of uMtCK elevated or reduced the HK2, PGK1 and LDHA mRNA levels, respectively. WB analysis further confirmed that HK2 expression was significantly increased or decreased upon uMtCK overexpression or knockdown, respectively; however, no significant changes were found for PGK1 and LDHA protein expression (Supplementary Fig. S3b). In the TCGA database, uMtCK expression was also positively associated with HK2 expression at the transcriptional levels (Supplementary Fig. S3c). These findings indicated that uMtCK may promotes glycolysis in GC cells in an HK2-dependent manner. Then, loss- and gain-of-function assays were applied by transfecting HK2 knockdown plasmids or overexpression vectors into uMtCK overexpressing or knockdown cells, respectively. As expected, knockdown or overexpression of HK2 in uMtCK overexpressing or knockdown cells partially reversed the increases or decreases in ATP production, glucose uptake and lactate production (Fig. 3h–m). Additionally, ECAR measurements suggested that knockdown or overexpression of HK2 evidently counteracted the strengthened or attenuated effect of uMtCK overexpression or knockdown on glycolytic capacity (Fig. 3n, o). As HK2 expression is closely related to the rate of fluoro-2-D-deoxyglucose F18 (18F-FDG) uptake in GC [21], we further analyzed the rates of 18F-FDG PET/CT of 30 GC patients with tumor relapse. The results displayed that the SUV max values in the uMtCK-high group (15.6 ± 9.1) were significantly higher than those in the uMtCK-low group (8.3 ± 6.0), indicating enhanced glucose metabolism in tumor tissues with uMtCK upregulation (Fig. 3p, q).

### uMtCK promotes GC cell glycolysis by activating the JNK-MAPK/JUN signaling pathway

**Fig. 4:**
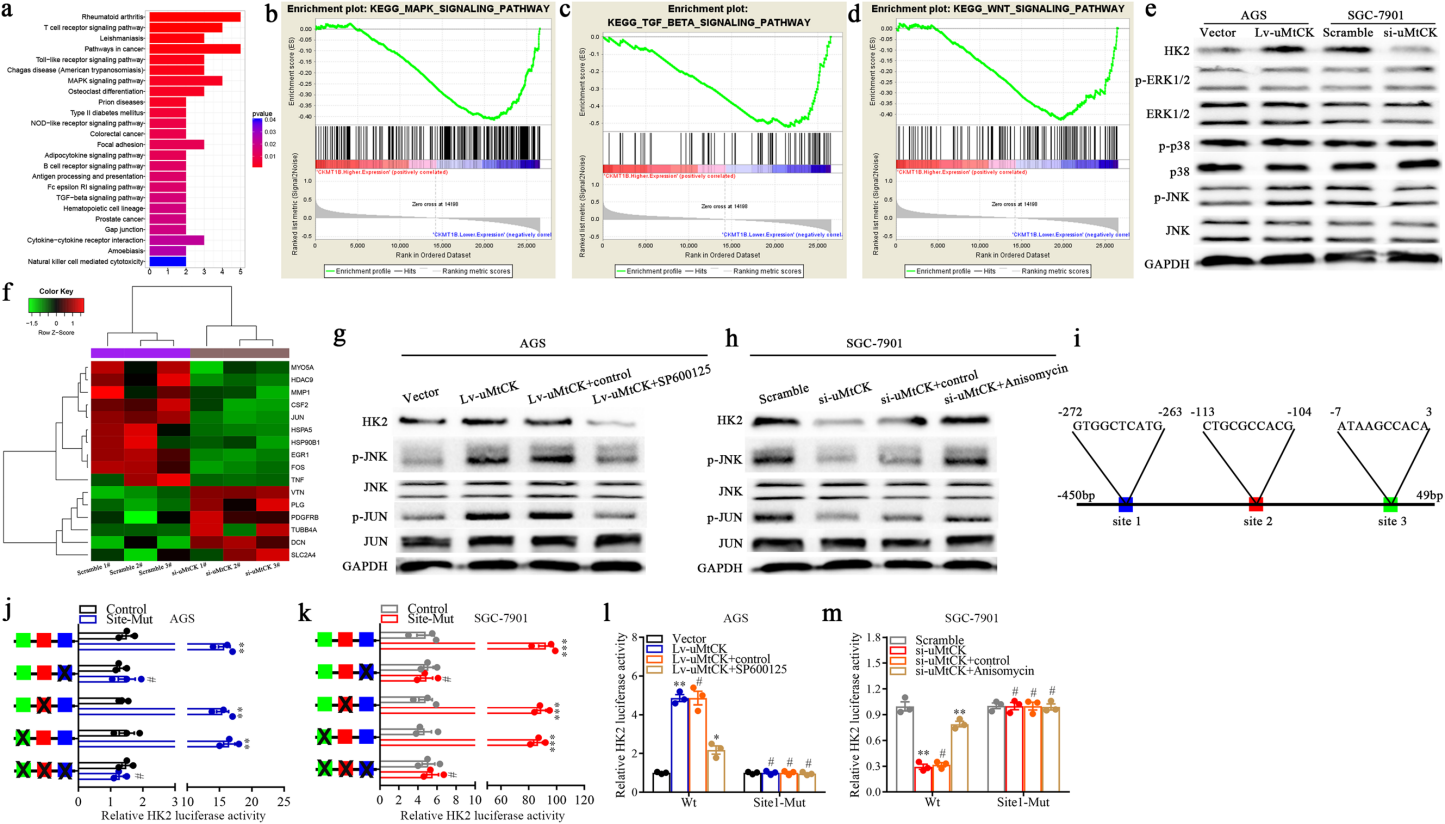
uMtCK promoted GC cell glycolysis through the JNK-MAPK/JUN/HK2 axis. **a** The pathways with significantly enriched up-regulated genes and down-regulated genes by RNA-sequencing through stable uMtCK knockdown or scramble control GC cells. The potential pathway of uMtCK (also known as CKMT1B) by a gene set enrichment analysis (GSEA) and MAPK (**b**), TGF-β (**c**) and WNT (**d**) were initially screened, showing a marked association with uMtCK aberrant expression. **e** The effect of uMtCK overexpression or knockdown on the expression of MAPK signaling pathway in GC cells AGS/Lv-uMtCK and SGC-7901/si-uMtCK as well as their control groups by WB assays, respectively. **f** The results of network key gene analysis showed that the samples could be completely clustered when the top 10 nodes with the highest score of each centrality method were taken and 16 genes were combined after the elimination of duplication. The effect of uMtCK overexpression or knockdown combined with HK2 knockdown or overexpression on the expression of HK2, JNK, p-JNK, JUN and p-JUN in GC cells AGS/Lv-uMtCK (**g**) and SGC-7901/si-uMtCK (**h**) as well as their control groups by WB assays, respectively. **i** Schematic diagram of the three specific binding sites between JUN and the promoter of HK2 based on JASPAR CORE database. **j**, **k** The binding site truncation mutants and their control vectors were cloned into pGL3-luciferase reporter plasmids and transfected into AGS/SGC-7901 cells. A luciferase reporter gene activity assay was used to analyze the changes in luciferase activity and determine the transcriptional sites. A luciferase reporter gene activity assay was further used to analyze the combined effects of JNK-MAPK/JUN signaling pathway inhibitors (SP600125) or activation agent (Anisomycin) on the luciferase activity of the wild-type/mutant JUN on binding site 1 of the HK2 promoter constructs in AGS/SGC-7901 cells. (*P < 0.05, **P < 0.01, ***P < 0.001, ^#^*P* > 0.05)

To reveal the mechanism of uMtCK promoting the progression of GC, we implemented RNA-sequencing through stable uMtCK knockdown or scramble control cells, the pathways with significantly enriched up-regulated genes and down-regulated genes are shown in Fig. 4a. Besides, we discovered that uMtCK knockdown was apparently associated with extracellular matrix (ECM) deposition and glycolysis (Supplementary Fig. S3d). Following KEGG analysis based on RNA-sequencing data (Fig. 4b–d), we preliminarily identified the MAPK signaling pathway as the key pathway upon uMtCK knockdown in GC cells. Next, our WB assays showed that uMtCK overexpression or knockdown, respectively, increases or decreased the expression of HK2 and the phosphorylation of JNK (p-JNK) but not ERK1/2 or p38 (Fig. 4e). The results of RNA-sequencing also displayed a total of 697 uMtCK-responsive genes, of which 385 were up-regulated (|logFC|> 1, *P* < 0.05) and 312 were down-regulated (|logFC|> 1, *P* < 0.05, Supplementary Fig. S3e, f). Further network key gene analysis showed that knockdown group and control group could be completely clustered when the top 10 nodes with the highest scores of each centrality method were taken and 16 genes were combined after the elimination of duplication (Fig. 4f). Among the 16 differentially expressed genes mentioned above, we selected 10 genes with the largest selection node degree, which displayed that FOS and JUN was significantly down-regulated after uMtCK knockdown. As reported, FOS encode leucine zipper proteins that can dimerize with proteins of the JUN, thereby forming the transcription factor complex, which serves as the downstream transcription factor after JNK-MAPK activation. As shown in Fig. 4g, h, uMtCK overexpression or knockdown in GC cells facilitated or inhibited the expression of HK2 and the phosphorylation of JNK as well as JUN, however, JNK-MAPK/JUN signaling pathway inhibitors (SP600125) or activation agent (Anisomycin) could partially offset the upper effect, respectively. Accordingly, we hypothesized that the activation of the JNK-MAPK signaling pathway may upregulate HK2 through the transcriptional function of JUN in GC. To confirm this hypothesis, we first predicted the potential transcriptional binding regions and specific binding sites using the tool the JASPAR CORE database. There were three specific binding sites between JUN and the promoter of HK2 (Supplementary Fig. S4). Subsequently, we constructed wild-type and mutant variants with the binding site mutated (Supplementary Table S9), and a luciferase reporter gene assay was used to confirm the transcriptional effect of JUN on the promoter of HK2 in AGS/SGC-7901 GC cells, respectively (Fig. 4i). We discovered that only mutant binding site 1 could downregulate luciferase reporter gene activity of HK2 and inhibit HK2 transcriptional expression (Fig. 4j, k). Moreover, we demonstrated that the promotive or suppressive role of uMtCK overexpression or knockdown on the expression of HK2 in GC cells could be apparently counteracted by treatment with JNK-MAPK/JUN signaling pathway inhibitors or activation agent, respectively; however, these processes were abolished when binding site 1 was mutated in GC cells (Fig. 4l, m).

### Altered JNK-MAPK/JUN signaling pathway ablate the promotive effect of uMtCK on glycolysis in GC cells

**Fig. 5:**
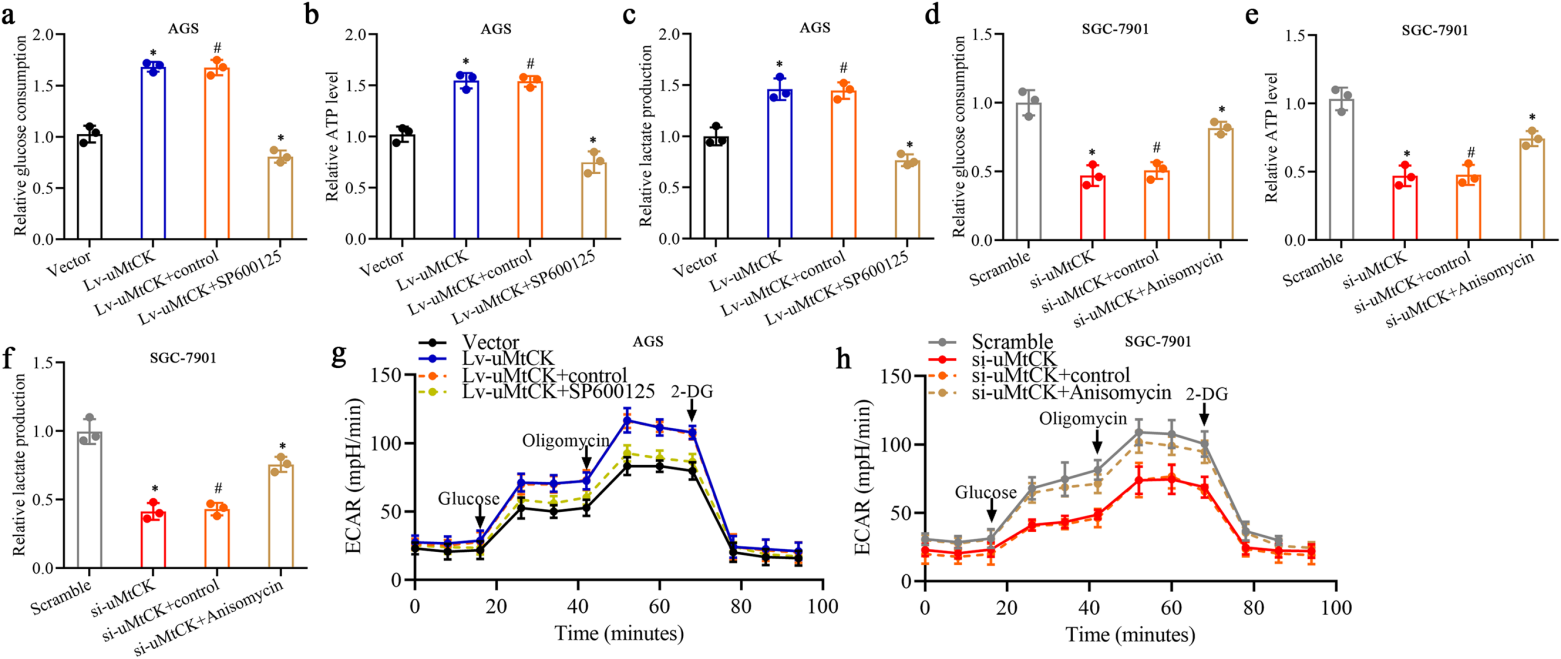
Altered JNK-MAPK/JUN signaling pathway ablate the promotive effect of uMtCK on glycolysis in GC cells. The effect of JNK-MAPK/JUN signaling pathway inhibitors (SP600125) or activation agent (Anisomycin) on the promotive or inhibitive role of uMtCK overexpression or knockdown on changes in relative glucose consumption (**a**, **c**), ATP levels (**b**, **d**), lactate production (**e**, **f**) and ECAR levels (**g**, **h**) in GC cells AGS/Lv-uMtCK and SGC-7901/si-uMtCK as well as their control groups, respectively. (*P < 0.05, ^#^*P* > 0.05)

To further investigate the impact of the JNK-MAPK/JUN pathway on the glycolysis regulated by uMtCK in GC cells, we first suppressed JNK-MAPK/JUN signaling pathway by added inhibitor of the JNK-MAPK/JUN signaling pathway (SP600125) in GC cells with stable overexpression of uMtCK. Glycolysis analysis indicated that restrained JNK-MAPK/JUN signaling pathway in GC cells with ectopic overexpression of uMtCK reversed the strengthened effect of uMtCK on ATP production, glucose uptake and lactate production (Fig. 5a–c). Then, we restored JNK-MAPK signaling pathway in uMtCK knockdown cells via appending JNK-MAPK/JUN signaling pathway activation agent (Anisomycin) in GC cells with uMtCK knockdown. As envisaged, restored JNK-MAPK/JUN signaling pathway in uMtCK knockdown cells renovated its enhancement on ATP production, glucose uptake and lactate production in GC cells (Fig. 5d–f). Similarly, the results of ECAR measurements showed that restrained or restored JNK-MAPK/JUN signaling pathway in GC cells significantly reversed the enhanced or receded effect of uMtCK overexpression or knockdown on glycolytic capacity (Fig. 5g, h). Collectively, these results suggest that uMtCK enhanced GC cells glycolysis by activating the JNK-MAPK/JUN signaling pathway.

### uMtCK overexpression facilitates GC cell metastasis in an HK2-dependent manner

**Fig. 6:**
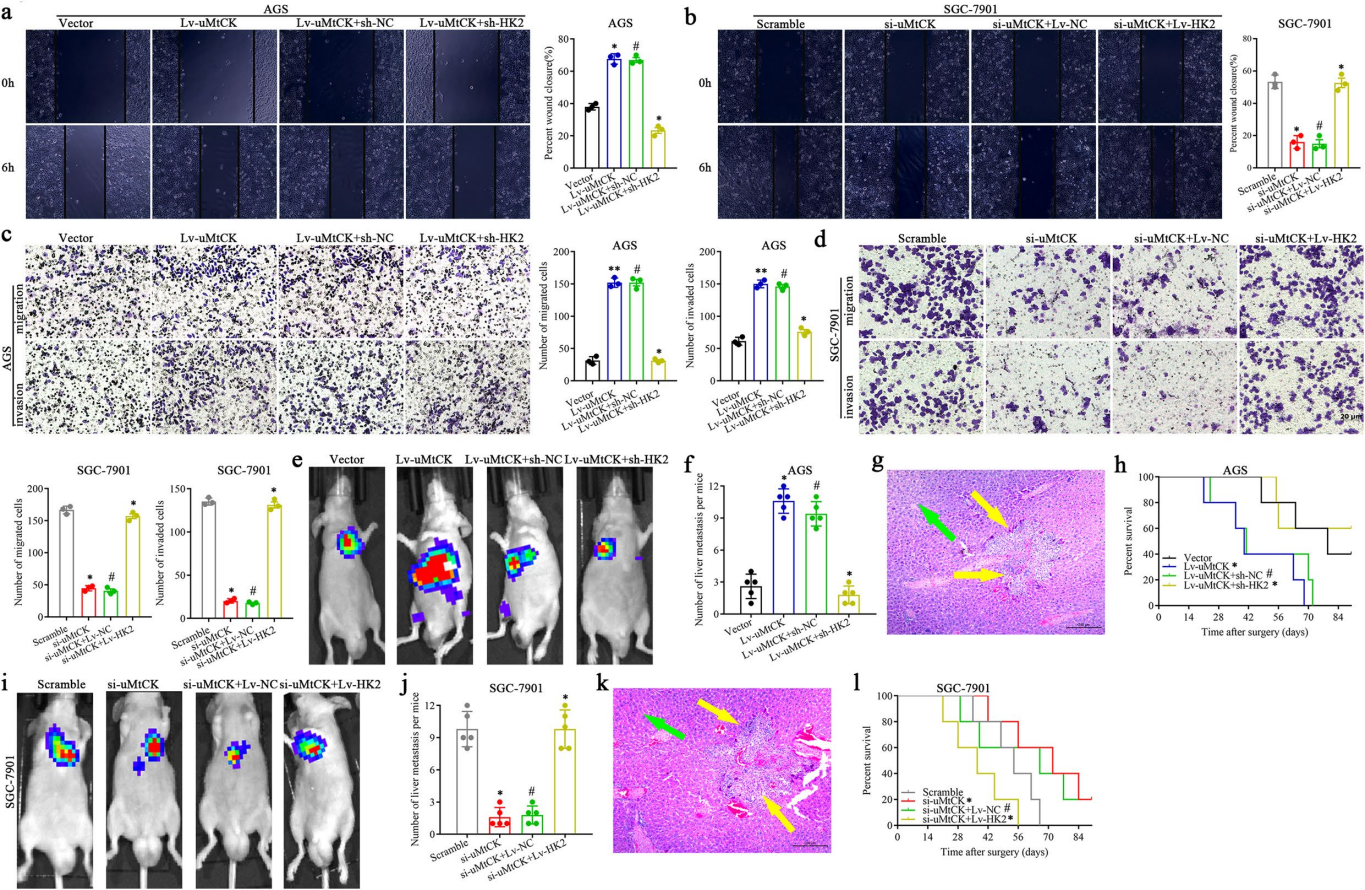
uMtCK facilitates GC cell migration, invasion in vitro and liver metastasis in vivo in an HK2-dependent manner. The effect of HK2 overexpression or knockdown on the promotive or inhibitive role uMtCK overexpression or knockdown on GC cells wound-healing (**a**, **b**), migration and invasion (**c**, **d**) compared with their control group, respectively. **e–l** The effect of HK2 overexpression or knockdown on the facilitated or receded role on the impact of uMtCK on GC cells liver metastasis in vivo. Representative formation of liver metastases by a spleen injection of AGS/Lv-uMtCK-luc and SGC-7901/si-uMtCK–Luc as well as their control group cells into nude mice, respectively. **e, i** Representative images of the luciferase signals (*n* = 5). The number of liver metastatic lesions was counted (*P < 0.05). **g**, **k** The images of the liver metastatic lesions by HE (green arrows, normal tissues; yellow arrows, liver metastatic lesions). **h**, **l** OS of each group of mice injected with engineered cells. (*P < 0.05, *P < 0.01, ^#^*P* > 0.05). uMtCK enhances the glycolysis of GC cells in an HK2-dependent manner and further promoted their migration, invasion and liver metastasis by activating the JNK-MAPK/JUN axis

Subsequently, we explored whether uMtCK could promote the malignant phenotype of GC cells in an HK2-dependent manner. We found that overexpression or knockdown of uMtCK accelerated or delayed GC cell wound-healing (Fig. 6a, b), as well as strengthened or attenuated GC cell migration and invasion in vitro, respectively (Fig. 6. 6c, d). Interestingly, the upper enhanced or suppressed wound-healing, migration and invasion upon overexpression or knockdown of uMtCK was markedly weakened by silencing HK2 or enhanced by overexpressing HK2 in GC cells, respectively (Fig. 6a–d). By transfecting HK2 knockdown plasmids or overexpression vectors into uMtCK overexpressing or knockdown cells, respectively, we further investigated whether the promotive role of uMtCK on GC liver metastasis in vivo would be affected by HK2. The results displayed that the promoted or inhibited impact of uMtCK overexpression or downregulation were evidently neutralized by HK2 knockdown or overexpression (Fig. 6e–l). In addition, the shortened or prolonged role of uMtCK overexpression or knockdown on OS time of nude mice was distinctly offset by HK2 knockdown or overexpression, respectively (Fig. 6h, l). Accordingly, these findings revealed that uMtCK also promoted GC cell migration and invasion in an HK2-dependent manner.

## Discussion

Due to the lack of effective early diagnosis technology, most GC patients are diagnosed at advanced stages, and metastasis comes to be known as the main cause of death in GC patients [2, 22]. According these, studies of prognostic factors for GC patients and of predictors for risk of recurrence and metastasis are urgently needed. In the present study, we revealed that uMtCK was aberrant overexpressed in GC and higher uMtCK expression predicted a poorer prognosis in GC patients, particularly for those with advanced UICC stage and tumor relapse. More importantly, uMtCK was proved to be an independent prognostic biomarker for GC patients. Although the present retrospective study only included 264 patients, our preliminary results provided strong evidence for prospective studies in the future.

uMtCK, a central controller of cellular energy homeostasis, is co-expressed with cytosolic CK in many cells, particularly in tissues with high-energy demands, such as the brain, placenta and endothelial cells [23], and its overexpression has been reported in certain malignant cancers with a poor prognosis, such as breast cancer [10, 11], liver cancer [12] and lung cancer [24]. Although a high-energy turnover is the main explanation that is generally accepted to explain uMtCK overexpression in cancers with a poor prognosis [25], the mechanism responsible for uMtCK overexpression in GC tumorigenesis and progression has not been elucidated. It will be helpful to study the specific and possible mechanisms of uMtCK promoting GC progression. Understandably, the metabolic switch from mitochondrial respiration to glycolysis during hypoxia as well as mitochondrial dysfunction are fundamental processes implicated in cancer metastasis [14, 26, 27]. Mitochondrial impairment or defective oxidative phosphorylation is frequently found in cancer and it is clear that mitochondrial defect in cancer cells can cause a shift in energy metabolism [14]. As uMtCK is known as a primary target of oxidative and radical-induced molecular damage and the impairment of uMtCK has been reported involved in induction of abnormal mitochondrial structure and a decrease of mitochondrial membrane potential, which may explain the mechanism of compensating for the functional impairment of the energy state control [28, 29]. In this study, we demonstrated that uMtCK could enhance glycolysis in GC cells and facilitated their migration, invasion and liver metastasis in vitro and in vivo.

Cancer cells exhibit aberrant metabolism characterized by high glycolysis even in the presence of abundant oxygen, known as the Warburg effect or aerobic glycolysis, facilitates tumor progression with elevated glucose uptake and lactate production [2, 13]. Recent investigations have shed light on the understanding of the benefits and selective advantages of aerobic glycolysis [13]. HK2 is a glycolytic enzyme that catalyzes the first committed step in glucose metabolism, and its upregulation has been observed in many types of cancer, promoting tumor metastasis and glycolysis as well as being a target of transcription factor in several cancers, which playing direct roles in regulation of the Warburg effect [30–32]. Besides, the MAPK signaling pathway acts as a key energy homeostasis regulator, essential for glycolysis in tumorigenesis [33]. In the present study, we verified that uMtCK enhances glycolysis in GC cells in an HK2-dependent manner and further promoted their migration, invasion and liver metastasis. Moreover, by combining results from RNA-sequencing and KEGG database, we proved that uMtCK increased the expression of HK2 by activating the JNK-MAPK/JUN axis in GC. In addition, using JNK-MAPK/JUN signaling pathway inhibitor or activation agent, we demonstrated that uMtCK-induced GC glycolysis and metastasis is due, at least in part, to the activation of the JNK-MAPK/JUN signaling pathway. Our results may be helpful to reveal the mechanisms of GC progression and also underline the importance of glycolysis on cancers.

In conclusion, this study provided critical insight into the clinical significance of the uMtCK in human GC, predicting poor survival in GC patients as an independent prognostic factor. Moreover, uMtCK enhances the glycolysis of GC cells in an HK2-dependent manner and further promoted their migration, invasion and liver metastasis by activating the JNK-MAPK/JUN axis. We propose that uMtCK overexpression might be a clinically useful, undesirable prognostic molecular biomarker for GC patients, particularly for GC patients with an advanced UICC stage and tumor recurrence.

### Supplementary Information

Below is the link to the electronic supplementary material.

Supplementary file1 (DOCX 1332 KB)
