# Correction: Ubiquitous mitochondrial creatine kinase promotes the progression of gastric cancer through a JNK-MAPK/JUN/HK2 axis regulated glycolysis

**DOI:** 10.1007/s10120-024-01490-w

**Published:** 2024-03-22

**Authors:** Yushuai Mi, Quanhui Li, Bingtian Liu, Dehai Wang, Ziping Liu, Tianshi Wang, Yuan Wang, Yifeng Zang, Yan Zhou, Yugang Wen, Yinlu Ding

**Affiliations:** 1 Department of Gastrointestinal Surgery, The Second Hospital, Cheeloo College of Medicine, Shandong University, No. 247 Beiyuan Street, Jinan, 250033 China; 2 Department of General Surgery, Shanghai General Hospital, School of Medicine, Shanghai Jiaotong University, 85 Wujin Road, Shanghai, 200080 China

**Correction: Gastric Cancer (2022) 26:69–81** 10.1007/s10120-022-01340-7

In this article, the scramble group of the invasion experiment in Fig. 2d, the Vector group of the migration experiment in Fig. 6c, the si-uMtCK + Lv-HK2 group of the invasion experiment in Fig. 6d and the experimental results of Lv-uMtCK + sh-HK2 in Fig. 6e were erroneously published; the Figs. 2 and 6 should have appeared as shown below.

**Fig. 2:**
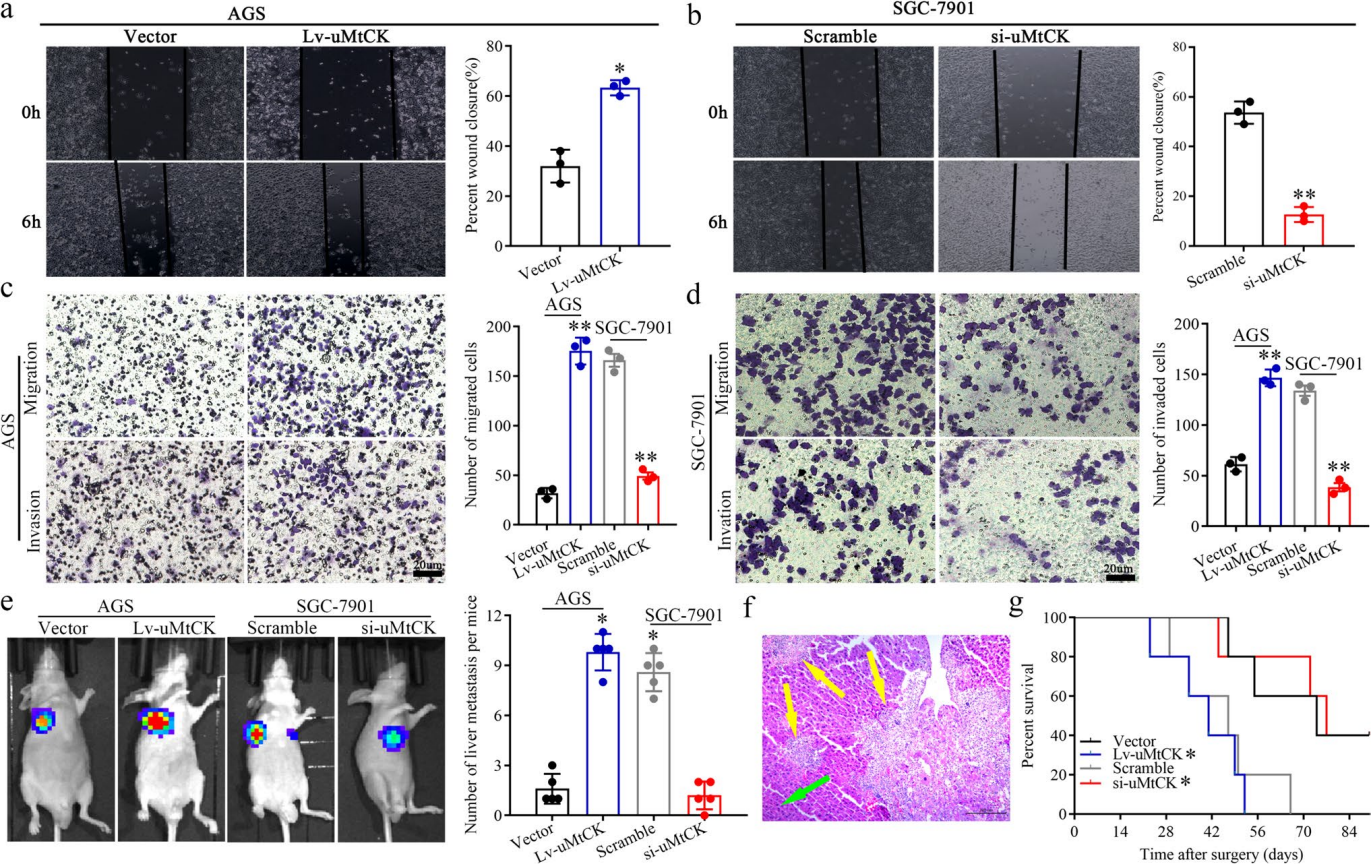
uMtCK facilitates GC cell migration, invasion in vitro and liver metastasis in vivo. uMtCK overexpression or knockdown increased or decreased GC wound-healing (**a**, **b**), migration and invasion (**c**, **d**) compared with their control group, respectively. **e–g** The impact of uMtCK on GC cells liver metastasis in vivo. Representative formation of liver metastases by a spleen injection of AGS/Lv-uMtCK-luc and SGC-7901/si-uMtCK–Luc as well as their control group cells into nude mice, respectively. **e** Representative images of the luciferase signals (*n* = 5). The number of liver metastatic lesions was counted (*P < 0.05). **f** The images of the liver metastatic lesions by HE (green arrows, normal tissues; yellow arrows, liver metastatic lesions). **g** OS of each group of mice injected with engineered cells. (*P < 0.05, *P < 0.01)

**Fig. 6:**
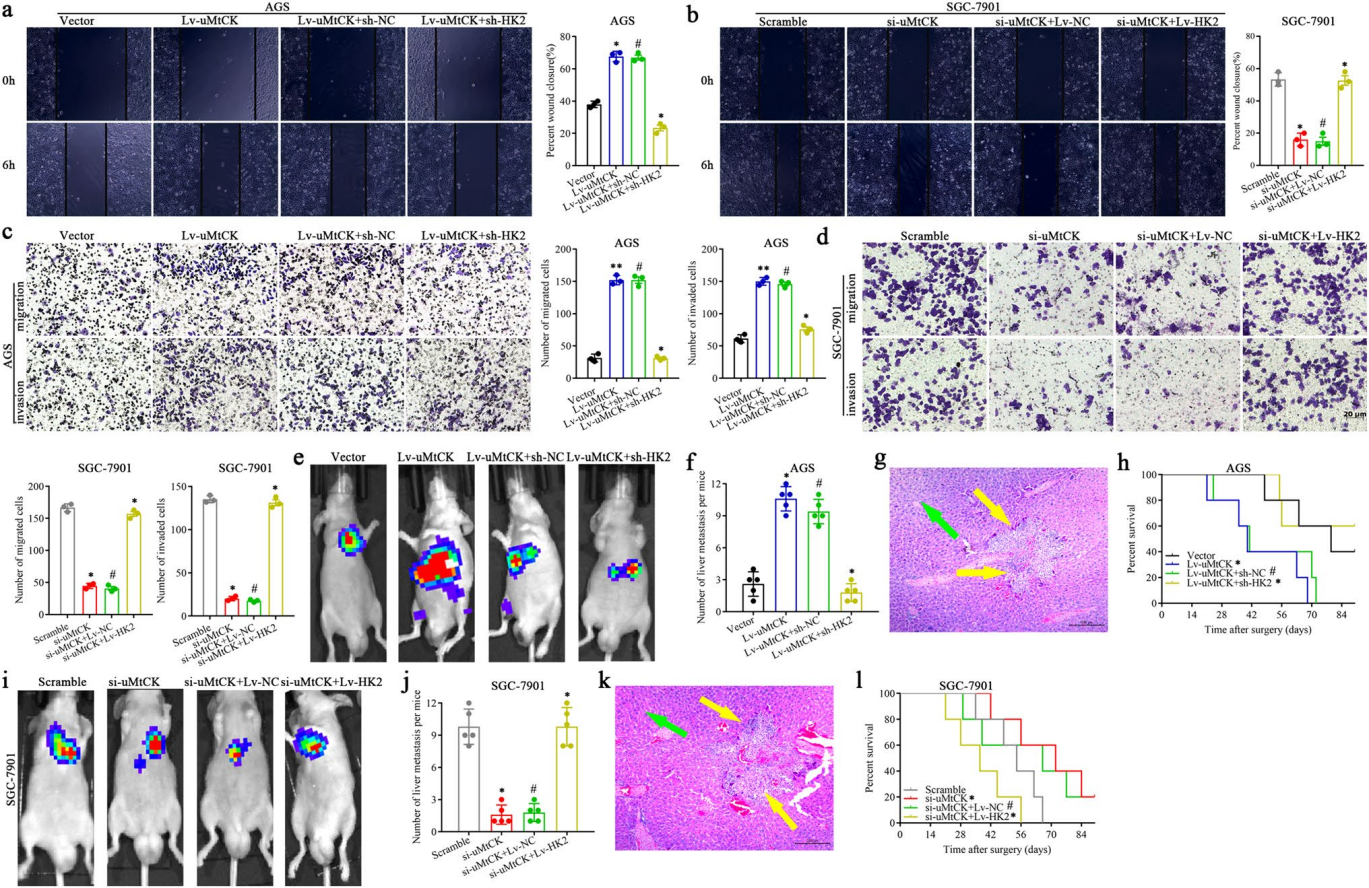
uMtCK facilitates GC cell migration, invasion in vitro and liver metastasis in vivo in an HK2-dependent manner. The effect of HK2 overexpression or knockdown on the promotive or inhibitive role uMtCK overexpression or knockdown on GC cells wound-healing (**a**, **b**), migration and invasion (**c**, **d**) compared with their control group, respectively. **e–l** The effect of HK2 overexpression or knockdown on the facilitated or receded role on the impact of uMtCK on GC cells liver metastasis in vivo. Representative formation of liver metastases by a spleen injection of AGS/Lv-uMtCK-luc and SGC-7901/si-uMtCK–Luc as well as their control group cells into nude mice, respectively. **e, i** Representative images of the luciferase signals (*n* = 5). The number of liver metastatic lesions was counted (*P < 0.05). **g**, **k** The images of the liver metastatic lesions by HE (green arrows, normal tissues; yellow arrows, liver metastatic lesions). **h**, **l** OS of each group of mice injected with engineered cells. (*P < 0.05, *P < 0.01, ^#^*P* > 0.05). uMtCK enhances the glycolysis of GC cells in an HK2-dependent manner and further promoted their migration, invasion and liver metastasis by activating the JNK-MAPK/JUN axis

