# Supplementary material for: Ubiquitous mitochondrial creatine kinase promotes the progression of gastric cancer through a JNK-MAPK/JUN/HK2 axis regulated glycolysis
Source: Gastric Cancer. 2022 Sep 16;26(1):69–81. doi: 10.1007/s10120-022-01340-7 (PMC9813075; doi:10.1007/s10120-022-01340-7)
Supplement: Supplementary file 1 — Supplementary file1 (DOCX 1332 KB) [file 10120_2022_1340_MOESM1_ESM.docx]

**Article title: Ubiquitous mitochondrial creatine kinase promotes the progression of gastric cancer through a JNK-MAPK/JUN/HK2 axis regulated glycolysis**

**Journal name: Gastric cancer**

Yushuai Mi^1^, Quanhui Li^1^, Bingtian Liu^1^, Dehai Wang^1^, Ziping Liu^1^, Tianshi Wang^1^, Yuan Wang^1^, Yifeng Zang^1^, Yan Zhou^1^, Yugang Wen^3^*, Yinlu Ding^1^*

**^*^ Corresponding authors:**

**Yinlu Ding**, Department of Gastrointestinal Surgery, The Second Hospital, Cheeloo College of Medicine, Shandong University, No. 247 Beiyuan Street, Jinan 250033, China;

Email: dingyinlu@126.com

**Supplementary materials and Methods**

**Patients and cell lines.**

A total of 264 patients who underwent surgery for GC between 2004 and 2009 by the same surgical team from Shanghai General Hospital, School of Medicine, Shanghai Jiaotong University were included in this study (Supplementary Table S1). Patients who underwent preoperative chemotherapy and/or radiotherapy were excluded from this study. This research was approved by the Ethics Committee of Shanghai General Hospital and the Second Hospital, Cheeloo College of Medicine, Shandong University. Written informed consent was obtained from all subjects, and they were subjected to close clinical follow-up observation. The disease-free survival (DFS) and overall survival (OS) rates were defined as the interval from the initial surgery to clinically or radiologically proven recurrence/ metastasis and death, respectively.

The established GC cell lines AGS, HGC-27, BGC-823, MKN-45, MGC-803, MKN-28, and SGC-7901 and the human normal gastric mucosa cell line, GES-1, were all obtained from the Type Culture Collection of the Chinese Academy of Sciences (Shanghai, China). All of the cell lines were cultured in RPMI 1640 medium supplemented with 10% FBS (GIBCO, Grand Island, NY, USA) under a humidified atmosphere containing 5% CO2 at 37°C.

**Tissue specimens.**

All patient-derived specimens were collected and archived under protocols approved by the institutional review boards of the Shanghai General Hospital at Shanghai Jiao Tong University. Frozen in liquid nitrogen or formalin-fixed, the paraffin-embedded cancer tissues and their paired adjacent non-neoplastic gastric mucosal tissues were collected immediately after surgical resection for subsequent RNA extraction or immunohistochemical (IHC) staining. All diagnoses were confirmed by at least two pathologists and clinical stages were classified according to the guidelines of NCCN2010. As there were 10 stage IV patients with distant metastasis who received palliative treatment, not curative resection, when performed prognostic analysis, we have already excluded patients with distant metastasis from this study to evaluate the prognostic value of uMtCK accurately. The institutional review boards of the Shanghai General Hospital approved this research.

**Quantitative Real-time PCR.**

Total RNA was isolated from primary tumor tissues, adjacent non-neoplastic gastric mucosal tissues, and cell culture using TRIzol reagent (TaKaRa, Japan) according to the manufacturer’s instructions. RevertAid™First Strand cDNA Synthesis Kit (Fermentas, USA) was used to reverse transcribe 2 ug of RNA according to the manufacturer’s recommendations. Quantitative Real-time PCR assays were performed with 4 μl of cDNA (1:10 dilution) and SYBR green (TaKaRa) in a total volume of 20 μl using the ABI 7900 Real-time PCR System (ABI, USA). Relative quantities (Δ cycle threshold (Ct) values) were obtained by normalizing to glyceraldehyde-3-phosphate dehydrogenase (GAPDH). Each PCR product was run in triplicate, and the relative mRNA level was calculated by 2-∆∆Ct. All primers used in this study are summarized in Supplementary Tables S2.

**Western blot (WB) analysis.**

Tissues and cell lysates were extracted using RIPA lysis buffer with the protease inhibitor phenylmethanesulfonyl fluoride (Beyotime Biotechnology, Jiangsu, China) Protein concentration was measured using the BCA protein assay kit (Beyotime Biotechnology) according to the manufacturer’s instructions. Equivalent amounts of protein (30 ug) were separated on 10% SDSPAGE gel and then transferred onto PVDF membranes (Millipore, Billeria, MA) using standard protocols. Membranes were blocked in 5% skim milk in TBST buffer for 1.5 h at room temperature, followed by incubation with primary antibodies at 4°C overnight.

After incubation with a secondary antibody for 2 h at room temperature, proteins were detected using ECL regent (Millipore, Billeria, MA). All antibodies used in this study are summarized in Supplementary Tables S3.

**Tissue microarray (TMA) construction and immunohistochemistry (IHC) staining.**

TMA construction was undertaken as reported previously[[1](#_ENREF_1), [2](#_ENREF_2)]. IHC staining for the tumor samples collected from the patients and the nude mice was performed as described previously[[1](#_ENREF_1)], and all of the corresponding primary antibodies are summarized in Supplementary Table S3. The staining intensity for uMtCK was scored as follows: 0 (negative); 1 (weak); 2 (moderate); and 3 (strong). The staining area was scored as follows: 0 (negative staining); 1 (less than 10% positive staining); 2 (10-50% positive staining); and 3 (50-100% positive staining). The sum of the staining intensity and the staining area scores provided the overall score, which was divided into three groups as follows: 0–2, negative expression; 3–4, weak expression; and 5–6, strong expression. Two investigators who were blinded to the patients’ prognoses evaluated the staining, and the sum of the staining intensity and the staining extent scores were used as the final staining score.

**GC cells transfections**.

For the cell functional assays, to generate an effective small interfering RNA (siRNA) specially targeting uMtCK, 3 siRNAs were first designed as shown in Supplementary Tables S2. The siRNA sequence 5’- GCACACCACGGATCTAGAT -3’ was identified as the best sequence and used to inhibit uMtCK expression. A scrambled siRNA sequence 5’-TTCTCCGAACGTGTCACGT-3’ was used as the negative control. At the same time, full-length human uMtCK was applied to generate the pcDNA3.1-uMtCK plasmid for the gene overexpression assay. All of these experiments were performed with the help of Biolink Biotechnology Co. (Shanghai, China). The GC cells were transfected using Lipofectamine2000 following the manufacturer’s instructions. The cells transfected with the Oligofectamine 2000 reagent alone were included as mock controls.

**Cell wound-healing assay.**

Transfected cells were trypsinized and seeded into 6-well plates (1.0 × 105 cells/ well). Upon reaching the exponential growth phase, cells were wounded by a sterile pipette tip and then washed with PBS. Images were captured at 0, 12, 24, and 48-h intervals, and wound widths were quantified and compared to baseline values.

**Transwell assay.**

The transwell 24-well Boyden chamber (Corning, USA) with 8.0 μm pore size polycarbonate membrane was used for the cell migration (without Matrigel) andm invasion assays (with Matrigel) assays according to the manufacturer’s protocol. Briefly, each group of cells (5 ×104/chamber) was plated in the upper chambers in 200 ul serum-free media for 36 h, while the bottom chambers contained 600 ul media supplemented with 10% fetal bovine serum (FBS) as a chemoattractant. Cells that migrated and invaded to the reverse side of chamber inserts were fixed by methyl alcohol and stained with 0.1% crystal violet. Experiments were carried out in triplicate.

**Animal experiments.**

The *in vivo* assay using nude mice was approved by the Institutional Animal Care and Use Committee of Shanghai General Hospital and the Second Hospital, Cheeloo College of Medicine, Shandong University. To further investigate the liver metastatic effect of uMtCK *in vivo*, we utilized a GC liver metastasis model in four-week-old male, specific pathogen-free BALB/C nude mice. A total of 2-5×10^5^ AGS/Lv-uMtCK and SGC-7901/si-uMtCK as well as their control group cells were transfected with lenti-LUC virus and injected into the spleen of the nude mice. In weekly intervals, anesthetized mice were injected i.p. with D-luciferin (150 mg/kg) and imaged 10 min after the injection using the IVIS Illumina System (Caliper Life Sciences). After 9 weeks, tumor metastases that had formed in the livers were observed and macroscopically examined for gross lesions as well as counted under a microscope via HE staining.

**Glucose uptake, lactate production and ATP production assays**

GC cells were seeded into a 96-well plate and were then analyzed using a colorimetric glucose uptake assay kit (AAT Bioquest, Sunnyvale, CA, USA) and a colorimetric L-lactate assay kit (AAT Bioquest, Sunnyvale, CA, USA) according to the manufacturer’s protocols, respectively. For the ATP assay, cells cultured in 6-well plates were lysed in 200 μL/well lysis buffer on ice and centrifuged at 4°C. The ATP content in the supernatant was determined using an enhanced ATP assay kit (Beyotime Biotechnology, Shanghai, China) in adherence to the recommended protocol. The content was normalized to the cell number.

**Extracellular acidification rate (ECAR) assay**

According to the manufacturer’s guidelines, ECAR was measured by a Seahorse XFe24 extracellular flux analyzer (Seahorse Bioscience, Billerica, MA, USA). In Brief, on the day before the ECAR assay, the sensors were submerged in the calibrant and placed in a non-CO2 37℃ incubator overnight for improved hydration. Cells were seeded into a 24-well plate, and medium was added to a final volume of 250μL/well after 5 hours. After overnight incubation, assay medium containing 2 mM glutamine was used to rinse cells two times and was added to a final volume of 500 μL/well. Then, cells were incubated in a non-CO2 37°C atmosphere for 1 hour prior to the assay. Glucose (10 mM), oligomycin (1 μM), and 2-DG (100 mM) were injected sequentially to measure the ECAR with 3 repeated tests after each injection. Each point represents the average of three independent samples.

**Luciferase reporter assay**.

Luciferase activity was assessed according to the Dual-Luciferase Reporter Assay protocol (Promega, Madison, WI, USA) using a Veritas™ 96-well Microplate Luminometer (Promega) with a substrate dispenser (Promega). Briefly, AGS/Lv-uMtCK and SGC-7901/si-uMtCK as well as their control group cells stable cells were seeded into 96-well plates and transfected with 20 ng of each luciferase reporter using Lipofectamine LTX 12 h later. Then, 24 h after the transfection, the firefly and Renilla luciferase activities were measured. The Renilla luciferase activities were used to normalize the transfection efficiency.

**Statistical analyses.**

For continuous variables, expressed as the median and inter-quartile range, data are compared using the Mann–Whitney U-test for two-group comparisons or the Kruskal–Wallis test for three groups. For categorical variables, expressed as the numerical count and percentage, data are compared using the Chi-square or Fisher’s exact test. Survival curves were plotted using the Kaplan-Meier method with the log-rank test employed to compare differences. The hazard ratio (HR), with a 95% confidence interval, in Cox proportional hazard regressions were applied to estimate the hazard risk of the individual factors DFS and OS. For all tests, a *P*-value < 0.05 was considered statistically significant. All data analysis we performed using the SPSS 22.0 statistical software package (SPSS, Chicago, IL, USA).

**References:**

1. Mi Y, Zhang D, Jiang W, Weng J, Zhou C, Huang K, et al. miR-181a-5p promotes the progression of gastric cancer via RASSF6-mediated MAPK signalling activation. Cancer Lett. 2017; 389: 11-22.

2. Wen Y, Wang Q, Zhou C, Yan D, Qiu G, Yang C, et al. Decreased expression of RASSF6 is a novel independent prognostic marker of a worse outcome in gastric cancer patients after curative surgery. Ann Surg Oncol. 2011; 18: 3858-67.

**Supplementary Table S1: Clinicopathologic characteristics of 264 gastric cancer patients.**

| Characteristics | N (%) |
| --- | --- |
| Age (yr) |  |
| <65 | 121 (45.8) |
| >=65 | 143 (54.2) |
| Gender |  |
| Male | 157 (59.5) |
| Female | 107 (40.5) |
| Tumor location |  |
| Gastric fundus | 11 (4.2) |
| Gastric corpus | 123 (46.6) |
| Pylorus | 130 (49.2) |
| Tumor size (cm) |  |
| <3 | 77 (29.2) |
| >=3 | 187 (70.8) |
| T stage |  |
| T 1 | 76 (28.8) |
| T 2 | 42 (15.9) |
| T 3 | 118 (44.7) |
| T 4 | 28 (10.6) |
| N stage |  |
| N 0 | 116 (43.9) |
| N 1 | 91 (34.5) |
| N 2 | 40 (15.2) |
| N 3 | 17 (6.4) |
| M stage |  |
| M 0 | 254 (96.2) |
| M 1 | 10 (3.8) |
| UICC stage |  |
| Ⅰ | 95 (36.0) |
| Ⅱ | 48 (18.2) |
| Ⅲ | 89 (33.7) |
| Ⅳ | 32 (12.1) |
| Vessel invasion |  |
| No | 186 (70.5) |
| Yes | 78 (29.5) |
| Nerve invasion |  |
| No | 206 (78.0) |
| Yes | 58 (22.0) |
| Differentiation |  |
| Well | 47 (17.8) |
| Moderate | 42 (15.9) |
| Poorly ^a^ | 175 (66.3) |
| Relapse |  |
| No | 145 (54.9) |
| Yes | 119 (45.1) |

^a^ Poorly differentiation corresponds to signet ring cell carcinoma, mucinous adenocarcinoma, and poorly differentiated adenocarcinoma

**Supplementary Table S2: Data of primer sequences used in this study.**

| **Gene** | **Primer sequence (5’-3’)** |
| --- | --- |
| uMtCK | Forward: AGA TAG CCG CTT CCC AAA GA  Reverse: CAA AGA CAC CGC CTG TAG CA |
| GAPDH | Forward: GGG AAG GTG AAG GTC GGA GT  Reverse: GGG GTC ATT GAT GGC AAC A |
| si-uMtCK-1# | Forward: GTGATCCAAGAGCGACACAAT  Reverse: ATTGTGTCGCTCTTGGATCAC |
| si-uMtCK-2# | Forward: GCTAAGCAAAGATAGCCGCTT  Reverse: AAGCGGCTATCTTTGCTTAGC |
| si-uMtCK-3# | Forward: GCACACCACGGATCTAGAT  Reverse: ATCTAGATCCGTGGTGTGC |
| GLUT1 | Forward: GCTTCCAGTATGTGGAGCAAC  Reverse: CCTGTGCTCCTGAGAGATCC |
| GLUT4 | Forward: ACTGGCCATTGTTATCGGCA  Reverse: GTCAGGCGCTTCAGACTCTT |
| HK1 | Forward: GAGTCTGGACGCGGGAATC  Reverse: CAGGTGGGCTCCTCATAAGC |
| HK2 | Forward: AAGGAGATGGAGAAAGGGCTTG  Reverse: AGCCAGGAACTCTCCGTGTT |
| PFK1 | Forward: GGTGCCCGTGTCTTCTTTGT  Reverse: AAGCATCATCGAAACGCTCTC |
| PGK1 | Forward: AGCGGGTCGTTATGAGAGTC  Reverse: CTCCAGCAGGATGACAGACC |
| PKM1 | Forward: CTATCCTCTGGAGGCTGTGC  Reverse: CCATGAGGTCTGTGGAGTGA |
| PKM2 | Forward: GGGTTCGGAGGTTTGATG  Reverse: ACGGCGGTGGCTTCTGT |
| LDHA | Forward: GCAGGTGGTTGAGAGTGCTTA  Reverse: CTTCAAACGGGCCTCTTCCT |
| LDHB | Forward: GAGCCTTCTCTCTCCTGTGC  Reverse: CAGCCAGAGACTTTCCCAGA |
| si-HK2 | Forward: CCAAAGACATCTCAGACATTG  Reverse: GGTTTCTGTAGAGTCTGTAAC |

**Supplementary Table S3:** Data of antibodies used in our research.

| **Antibody** | **WB** | **IHC** | **Specificity** | **Company** |
| --- | --- | --- | --- | --- |
| uMtCK (ab204114) | 1:400 | 1:200 | Rabbit polyclonal | Abcam |
| GAPDH (60004-1-lg) | 1:1000 | - | Mouse monoclonal | Proteintech |
| HK2 (ab209847) | 1:600 | - | Rabbit monoclonal | Abcam |
| p-ERK1/2 (#9101) | 1:1000 | - | Rabbit polyclonal | Cell Signaling Technology |
| ERK1/2 (#9102) | 1:1000 | - | Rabbit polyclonal | Cell Signaling Technology |
| p-p38 (#9216) | 1:2000 | - | Mouse monoclonal | Cell Signaling Technology |
| P38 (#8690) | 1:1000 | - | Rabbit monoclonal | Cell Signaling Technology |
| p-JNK (#9255) | 1:2000 | - | Mouse monoclonal | Cell Signaling Technology |
| JNK (#9252) | 1:1000 | - | Rabbit polyclonal | Cell Signaling Technology |
| p-JUN (ab32385) | 1:1000 | - | Rabbit monoclonal | Abcam |
| JUN (ab40766) | 1:1000 | - | Rabbit monoclonal | Abcam |

**Supplementary Table S4. Association between uMtCK expression and the clinicopathological features in gastric cancer (n=40).**

|  | N |
| --- | --- |
|  |  |
| Age (yr) |  |
| <65 | 20 |
| >=65 | 20 |
| Gender |  |
| Male | 23 |
| Female | 17 |
| Tumor location |  |
| Gastric fundus | 20 |
| Gastric corpus | 20 |
| Pylorus | 0 |
| Tumor size (cm) |  |
| <3 | 5 |
| >=3 | 35 |
| T stage |  |
| T 1 | 3 |
| T 2 | 5 |
| T 3 | 20 |
| T 4 | 12 |
| N stage |  |
| N 0 | 12 |
| N 1 | 17 |
| N 2 | 7 |
| N 3 | 4 |
| M stage |  |
| M 0 | 37 |
| M 1 | 3 |
| UICC stage |  |
| Ⅰ | 5 |
| Ⅱ | 9 |
| Ⅲ | 17 |
| Ⅳ | 9 |
| Vessel invasion |  |
| No | 29 |
| Yes | 11 |
| Nerve invasion |  |
| No | 23 |
| Yes | 17 |
| Differentiation |  |
| Well | 4 |
| Moderate | 10 |
| Poorly ^a^ | 26 |
| Relapse |  |
| No | 12 |
| Yes | 28 |

^a^ Poorly differentiation corresponds to signet ring cell carcinoma, mucinous adenocarcinoma, and poorly differentiated adenocarcinoma

**Supplementary Table S5. uMtCK expression in non-neoplastic gastric mucosal tissues, primary cancerous tissues and lymph node metastasis (n=264).**

| Tissue sample | N | Expression of uMtCK | | | *P* |
| --- | --- | --- | --- | --- | --- |
|  | | Negative (%) | Weak positive (%) | Strong positive (%) |  |
| NGM tissues | 264 | 207 (78.4) | 44 (16.7) | 13 (4.9) | <0.001^a^ |
| GC tissue | 264 | 25 (9.5) | 93 (35.2) | 146 (55.3) | <0.001^b^ |
| LNM tissue | 104 | 5 (4.8) | 12 (11.5) | 87 (83.7) | 0.038^c^ |

NGM: non-neoplastic gastric mucosal tissues; GC: gastric cancer; LNM: lymph node metastasis

a Significant difference in the expression of uMtCK between non-neoplastic gastric mucosal tissues and cancerous tissues;

b Significant difference between GC tissues and LNM;

c Significant difference between LNM and non-neoplastic gastric mucosal tissues.

**Supplementary Table S6. Association between uMtCK expression and the clinicopathological features in gastric cancer (n=264).**

|  | N | uMtCK expression | | | *P* |
| --- | --- | --- | --- | --- | --- |
|  |  | Negative (25) | Weak positive (93) | Strong positive (146) |  |
| Age (yr) |  |  |  |  | 0.352 |
| <65 | 121 | 14 (11.6%) | 38 (31.4) | 69 (57.0%) |  |
| >=65 | 143 | 11 (7.7%) | 55 (38.5) | 77 (53.8%) |  |
| Gender |  |  |  |  | 0.327 |
| Male | 157 | 14 (8.9%) | 61 (38.9) | 82 (52.2%) |  |
| Female | 107 | 11 (10.3%) | 32 (29.9) | 64 (59.8%) |  |
| Tumor location |  |  |  |  | 0.485 |
| Gastric fundus | 11 | 0 (0.0%) | 6 (54.5%) | 5 (45.5%) |  |
| Gastric corpus | 123 | 10 (8.1%) | 42 (34.1%) | 71 (57.7%) |  |
| Pylorus | 130 | 15 (11.5%) | 45 (34.6%) | 70 (53.8%) |  |
| Tumor size (cm) |  |  |  |  | 0.229 |
| <3 | 77 | 11 (14.3%) | 26 (33.8%) | 40 (51.9%) |  |
| >=3 | 187 | 14 (7.5%) | 67 (35.8%) | 106 (56.7%) |  |
| T stage |  |  |  |  | 0.03* |
| T 1 | 76 | 14 (18.4%) | 24 (31.6%) | 38 (50.0%) |  |
| T 2 | 42 | 3 (7.1%) | 18 (42.9%) | 21 (50.0%) |  |
| T 3 | 118 | 6 (5.1%) | 45 (38.1%) | 67 (56.8%) |  |
| T 4 | 28 | 2 (7.1%) | 6 (21.4%) | 20 (71.4%) |  |
| N stage |  |  |  |  | <0.001* |
| N 0 | 116 | 20 (17.2%) | 41 (35.3%) | 55 (47.4%) |  |
| N 1 | 91 | 2 (2.2%) | 40 (44.0%) | 49 (53.8%) |  |
| N 2 | 40 | 3 (7.5%) | 8 (20.0%) | 29 (72.5%) |  |
| N 3 | 17 | 0 (0.0%) | 4 (23.5%) | 13 (76.5%) |  |
| M stage |  |  |  |  | 0.015* |
| M 0 | 254 | 25 (9.8%) | 93 (36.6%) | 136 (53.5%) |  |
| M 1 | 10 | 0 (0.0%) | 0 (0.0%) | 10 (100.0%) |  |
| UICC stage |  |  |  |  | <0.001* |
| Ⅰ | 95 | 15 (15.8%) | 32 (33.7%) | 48 (50.5%) |  |
| Ⅱ | 48 | 7 (14.6%) | 25 (52.1%) | 16 (33.3%) |  |
| Ⅲ | 89 | 2 (2.2%) | 32 (36.0%) | 55 (61.8%) |  |
| Ⅳ | 32 | 1 (3.1%) | 4 (12.5%) | 27 (84.4%) |  |
| Vessel invasion |  |  |  |  | 0.428 |
| No | 186 | 20 (10.8%) | 67 (36.0%) | 99 (53.2%) |  |
| Yes | 78 | 5 (6.4%) | 26 (33.3%) | 47 (60.3%) |  |
| Nerve invasion |  |  |  |  | 0.681 |
| No | 206 | 20 (9.7%) | 75 (36.4%) | 111 (53.9%) |  |
| Yes | 58 | 5 (8.6%) | 18 (31.0%) | 35 (60.3%) |  |
| Differentiation |  |  |  |  | <0.001* |
| Well | 47 | 15 (31.9%) | 14 (29.8%) | 18 (38.3%) |  |
| Moderate | 42 | 1 (2.4%) | 18 (42.9%) | 23 (54.8%) |  |
| Poorly ^a^ | 175 | 9 (5.1%) | 61 (34.9%) | 105 (60.0%) |  |
| Relapse |  |  |  |  | <0.001* |
| No | 145 | 21 (14.5%) | 68 (46.9%) | 56 (38.6%) |  |
| Yes | 119 | 4 (3.4%) | 25 (21.0%) | 90 (75.6%) |  |

^a^ Poorly differentiation corresponds to signet ring cell carcinoma, mucinous adenocarcinoma, and poorly differentiated adenocarcinoma;

* Significant difference.

|  | Overall survival | | | | |
| --- | --- | --- | --- | --- | --- |
|  | Univariate analysis | |  | Multivariate analysis | |
|  | HR (95%CI) | *P* |  | HR (95%CI) | *P* |
| Age (yr) | 1.523 (0.947-2.450) | 0.083 |  |  |  |
| Gender | 1.061 (0.665-1.692) | 0.804 |  |  |  |
| Tumor location | 0.952 (0.635-1.426) | 0.811 |  |  |  |
| Tumor size (cm) | 1.370 (0.811-2.314) | 0.239 |  |  |  |
| T stage | 1.571 (1.225-2.014) | <0.001* |  | 1.220 (0.831-1.791) | 0.310 |
| N stage | 2.064 (1.634-2.608) | <0.001* |  | 1.303 (0.880-1.929) | 0.186 |
| M stage | 3.036 (1.314-7.014) | 0.009* |  | 2.133 (0.738-6.163) | 0.031* |
| UICC stage | 2.023 (1.590-2.575) | <0.001* |  | 1.114 (0.660-1.878) | 0.016* |
| Vessel invasion | 5.433 (3.368-8.763) | 0.079 |  |  |  |
| Nerve invasion | 3.136 (1.929-5.097) | 0.286 |  |  |  |
| Differentiation | 1.301 (0.932-1.816) | 0.122 |  |  |  |
| Relapse | 10.234 (5.579-18.774) | <0.001* |  | 8.359 (4.421-15.805) | <0.001* |
| uMtCK | 3.185 (1.964-5.166) | <0.001* |  | 2.026 (1.218-3.372) | 0.007* |

**Supplementary Table S7. Univariate and multivariate Cox proportional hazard models for overall survival after surgery (n=254).**

HR hazard ratio; CI confidence interval;

* P<0.05 indicate that the 95% CI of HR was not including 1.

**Supplementary Table S8. Univariate and multivariate Cox proportional hazard models for disease-free survival after surgery (n=254).**

HR hazard ratio; CI confidence interval;

* P<0.05 indicate that the 95% CI of HR was not including 1.

| Disease-free survival | | | | | |
| --- | --- | --- | --- | --- | --- |
| Univariate analysis | | |  | Multivariate analysis | |
| HR (95%CI) | | *P* |  | HR (95%CI) |  |
| Age (yr) | 1.152 (0.940-2.432) | 0.088 |  |  |  |
| Gender | 1.048 (0.657-1.670) | 0.845 |  |  |  |
| Tumor location | 0.946 (0.632-1.415) | 0.787 |  |  |  |
| Tumor size (cm) | 1.362 (0.806-2.299) | 0.248 |  |  |  |
| T stage | 1.567 (1.223-2.007) | <0.001* |  | 1.085 (0.737-1.597) | 0.680 |
| N stage | 2.112 (1.668-2.675) | <0.001* |  | 1.230 (0.806-1.875) | 0.337 |
| M stage | 3.555 (1.539-8.216) | 0.003* |  | 2.525 (0.924-6.900) | 0.044* |
| UICC stage | 2.081 (1.630-2.658) | <0.001* |  | 1.322 (0.765-2.283) | 0.024* |
| Vessel invasion | 5.653 (3.506-9.117) | 0.094 |  |  |  |
| Nerve invasion | 3.023 (1.862-4.910) | 0.359 |  |  |  |
| Differentiation | 1.307 (0,936-1.825) | 0.116 |  |  |  |
| Relapse | 10.298 (5.606-18.917) | <0.001* |  | 8.989 (4.711-17.154) | <0.001* |
| uMtCK | 3.150 (1.942-5.108) | <0.001* |  | 2.018 (1.213-3.357) | 0.007* |

**Supplementary Table S9: The wild (WT) and mutant (MUT) sequences of binding sites between JUN and the promoter of HK2.**

|  | **Stat** | **End** | **WT** | **MUT** |
| --- | --- | --- | --- | --- |
| 1 | -272 | -262 | GTGGCTCATG | ACAATCTGCA |
| 2 | -113 | -104 | CTGCGCCACG | TCATATTGTA |
| 3 | -7 | 3 | ATAAGCCACA | GCGGATTGTG |

**
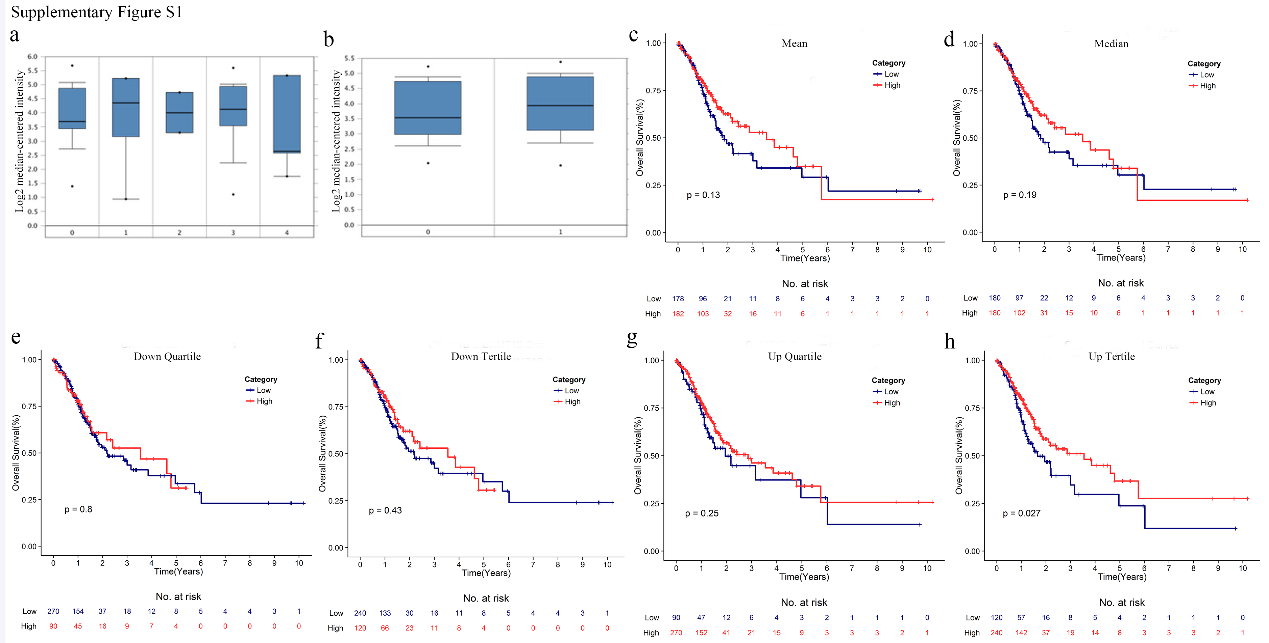
**

**
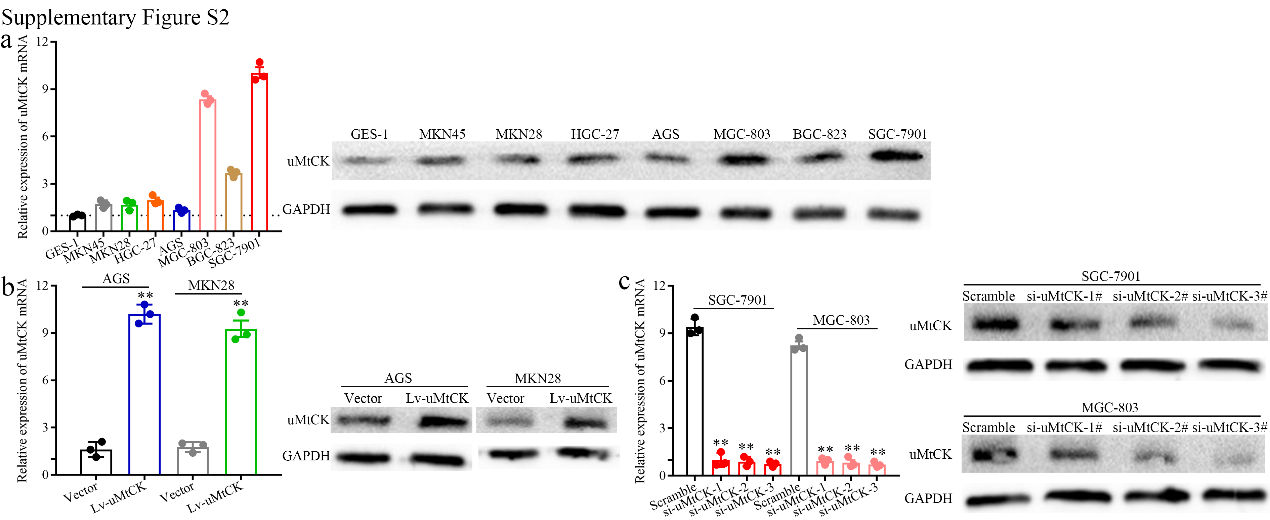
**

**
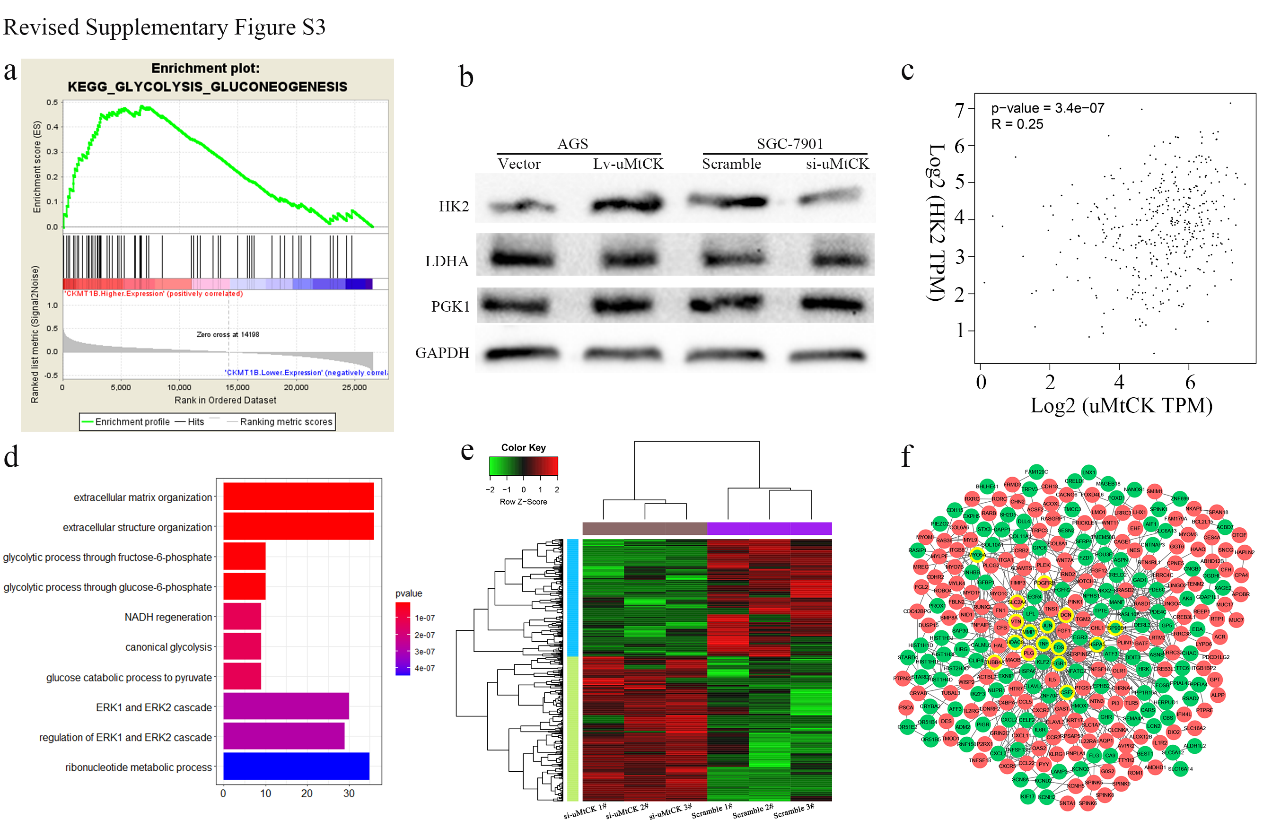
**

**
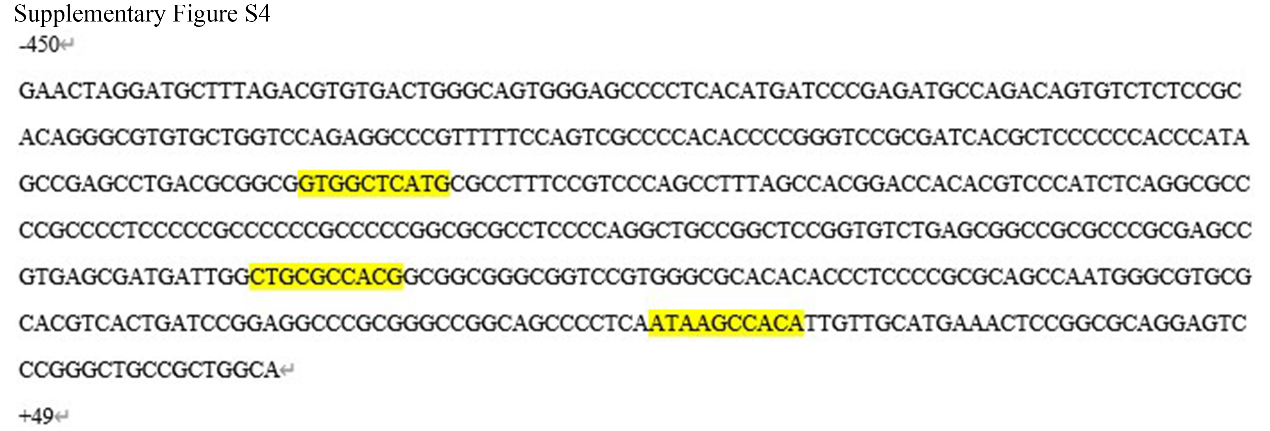
**

**Supplementary figure legends:**

**Supplementary Fig. S1 The expression levels of uMtCK in GC database and its prognostic role for GC patients at the mRNA level. a.** uMtCK expression at the mRNA level in DErrico Gastric grouped by normal gastric mucosa (0), diffuse gastric adenocarcinoma (1), gastric adenocarcinoma (2), gastric intestinal type adenocarcinoma (3) and gastric mixed adenocarcinoma (4) **b.** uMtCK expression at mRNA level in Wang Gastric grouped by normal gastric mucosa (0) and gastric cancer (1). The impact of uMtCK expression at the RNA level on GC patient overall survival (OS) in TCGA public databases. Significance between the aberrant expression of uMtCK at the RNA level and the OS was observed in the GC patients only at the upper-tertile classification. **c.** The mean RNA expression of uMtCK and the OS; **d.** The median RNA expression of uMtCK and the OS; **e.** The down-quartile RNA expression of uMtCK and the OS; **f.** The down-tertile RNA expression of uMtCK and the OS; **g.** The up-quartile RNA expression of uMtCK and the OS; **h.** The up-tertile RNA expression of uMtCK and the OS (*P*=0.027). (as a supplement to Fig. 1).

**Supplementary Fig. S2. The expression levels of uMtCK expression in gastric cell lines.** **a.** The expression levels of uMtCK in one healthy human gastric mucosa cell line and 7 GC cell lines at the mRNA and protein level were detected by qPCR and WB assays, respectively. Small interfering RNA (siRNA 1#, 2#, 3#) specially targeting uMtCK and pcDNA3.1-uMtCK plasmid for gene overexpression were transfected in AGS/MKN28 cells (**b**), which showed the lower uMtCK expression and SGC-7901/MGC-803 cells (**c**), which had the higher uMtCK expression, with their efficiencies were confirmed by qPCR and WB, respectively (***P*<0.01). (as a supplement to Fig. 2).

**Supplementary Fig. S3. a.** Gene set enrichment analysis (GSEA) for gene signatures of glycolysis/gluconeogenesis in GC cells with uMtCK (also known as CKMT1B) knockdown. **b.** The effect of uMtCK overexpression or knockdown on the expression of HK2 in GC cells AGS/Lv-uMtCK and SGC-7901/si-uMtCK as well as their control groups by WB assays, respectively. (as a supplement to Fig. 3). **c.** Correlation analysis by Spearman’s rank correlation coefficient (GEPIA, http://gepia.cancer-pku.cn/) showed the correlation within uMtCK and HK2 in the GC tissue specimens. **d.** Correlation between uMtCK knockdown and specific biological process in GC cells. **e.** Heatmap of RNA-sequencing in SGC-7901 GC cells transfected with uMtCK-shRNA1#, 2#, 3# or scramble groups. Red color indicates up-regulated genes and green color indicates down-regulated genes. **f.** STRING database was used to predict whether there was interaction between proteins encoded by differentially expressed genes. STRING database prediction methods come from Neighborhood, Gene Fusion, co-occurrence, co-expression Experiments, Databases, and Textmining. The input gene set is a differentially expressed gene and the species is Homo. PPI Score was set as 0.4 (Medium confidence), and all protein nodes interacting were required to be differentially expressed genes. (as a supplement to Fig. 4).

**Figure S4.** **Potential transcriptional binding sequences of JUN on the promoter of HK2 (ID:406954)**. The sequence of HK2 and the transcription start site are marked in black. The sequences marked in yellow are the three potential transcriptional binding sites of JUN on the promoter of HK2. (as a supplement to Fig. 4).
